# Editorial: Retinal vascular functional assessment in health and disease

**DOI:** 10.3389/fnins.2022.1008112

**Published:** 2022-09-07

**Authors:** Andrea Servillo, Riccardo Sacconi, Giuseppe Querques

**Affiliations:** ^1^Ophtalmology Unit, Division of Head and Neck, IRCSS Ospedale San Raffaele, Milan, Italy; ^2^School of Medicine, Vita-Salute San Raffaele University, Milan, Italy

**Keywords:** retina, psoriasis, myopia, diabetic retinopathy, OCTA

Optimal retinal neuronal cell function requires correctly regulated hemodynamics. Retinal vascular impairment is present in several blinding diseases affecting the population worldwide. The aim of this Research Topic was to collect translational studies related to the assessment of retinal vascularization.

Recently, optical coherence tomography angiography (OCTA) has significantly improved vascular assessment of the retina (Spaide et al., 2018). OCTA can identify early retinal vascular impairment even in the absence of clinical retinal and choroidal damage (Carnevali et al., 2017).

In this direction, Castellino et al. investigated the vascular status of the macula in psoriasis patients without history of ocular inflammation by OCTA since no data were available in current literature on the assessment of retinal vasculature by OCTA in psoriasis of patients. They found that alteration of vessel density (VD) may suggest that vascular changes may occur earlier than the clinical onset of posterior inflammation Castellino et al..

Pierro et al. focused on retinal and choroidal vascular perfusion changes before and after endarterectomy in patients affected by carotid arterial stenosis (CAS). The authors underlined the importance of the quantitative OCTA analyses in detecting retinal perfusion changes even in asymptomatic CAS patients (Pierro et al.). Future investigations on CAS patients could also provide quantitative cut-off values to evaluate the risk of ocular involvement and to develop clinically helpful prognostic biomarkers in CAS (Pierro et al.).

Therefore, OCTA has significantly improved our capability to investigate and quantify retinal perfusion, allowing a quantitative analysis of several parameters of retinal vasculature, including foveal avascular zone size, perfusion density, vessel length density, vessel diameter index, and fractal dimension. However, currently, there is no established metric for the detection of vessel closure (VC) (Mendes et al.). Mendes et al. compared the performance of 11 different metrics, based on OCTA, for the identification of VC in different Early Treatment for Diabetic Retinopathy Study (ETDRS) severity groups. They reported that the values of these metrics on the different ETDRS groups showed a progressive increase in VC, which was correlated with disease severity (Mendes et al.).

Furthermore, in addition to its diagnostic and clinical management role, OCTA may represent an important tool to contribute to the pathophysiological knowledge of retinal changes in systemic and ocular diseases. Regarding this point, here three studies analyzed the characteristic of retinal vasculature on optical coherence tomography (OCT) and OCTA in high myopic patients.

Li et al. investigated the characteristics of macular structures and microcirculation on OCT/OCTA of posterior staphyloma (PS) and explored factors related to PS in eyes with high myopia. They found that subfoveal retinal thickness (SFST), choroidal thickness (CT), and choriocapillaris perfusion area (CCPA) were significantly correlated with PS, concluding that PS was correlated with abnormalities in macular structures and microcirculation (Li et al.).

Moreover, Xu et al. evaluated the use of OCTA in patients diagnosed with high myopia and undergoing implantable Collamer lens surgery to more comprehensively evaluate postoperative retinal circulation parameters and explore the influence of their changes on the occurrence and development of pathological myopia. After surgery, the changes in retinal vascular density in patients with high myopia were more significant than those in normal populations (Xu et al.).

Finally, Wei et al. explored the macular structures and vascular characteristics of more myopic (MM) and contralateral eyes with highly myopic anisometropia on OCT/OCTA, demonstrating that retinal and choroidal layers tended to be thinner in MM eyes.

The impairment of retinal blood flow in psoriasis, carotid artery stenosis, diabetes and myopia affects the vision of millions of people worldwide. These published articles on retinal vascular functional assessment may impact the current and the future knowledge on several important diseases affecting the complex system of retinal vasculature. For this reason, we truly believe that the findings showed in this Research Topic may improve the knowledge on innovative ways and techniques for the assessment of retinal vascular behavior in different diseases.

## Author contributions

All authors listed have made a substantial, direct, and intellectual contribution to the work and approved it for publication.

## Conflict of interest

RS received financial support from Allergan Inc, Bayer Shering-Pharma, Medivis, Novartis, and Zeiss. GQ received financial support from Alimera Sciences, Allergan Inc, Amgen, Bayer Shering-Pharma, Heidelberg, KBH, LEH Pharma, Lumithera, Novartis, Sandoz, Sifi, Sooft-Fidea, and Zeiss. The funder was not involved in the study design, collection, analysis, interpretation of data, the writing of this article or the decision to submit it for publication. The remaining author declares that the research was conducted in the absence of any commercial or financial relationships that could be construed as a potential conflict of interest.

## Publisher's note

All claims expressed in this article are solely those of the authors and do not necessarily represent those of their affiliated organizations, or those of the publisher, the editors and the reviewers. Any product that may be evaluated in this article, or claim that may be made by its manufacturer, is not guaranteed or endorsed by the publisher.
